# Peptide TaY Attenuates Inflammatory Responses by Interacting with Myeloid Differentiation 2 and Inhibiting NF-κB Signaling Pathway

**DOI:** 10.3390/molecules29204843

**Published:** 2024-10-13

**Authors:** Junyong Wang, Yichen Zhou, Jing Zhang, Yucui Tong, Zaheer Abbas, Xuelian Zhao, Zhenzhen Li, Haosen Zhang, Sichao Chen, Dayong Si, Rijun Zhang, Xubiao Wei

**Affiliations:** Laboratory of Feed Biotechnology, State Key Laboratory of Animal Nutrition and Feeding, College of Animal Science and Technology, China Agricultural University, Beijing 100193, China

**Keywords:** peptides, TLR4/MD2, LPS, anti-inflammatory, RAW 264.7

## Abstract

A balanced inflammatory response is crucial for the organism to defend against external infections, however, an exaggerated response may lead to detrimental effects, including tissue damage and even the onset of disease. Therefore, anti-inflammatory drugs are essential for the rational control of inflammation. In this study, we found that a previously screened peptide TaY (KEKKEVVEYGPSSYGYG) was able to inhibit the LPS-induced RAW264.7 inflammatory response by decreasing a series of proinflammatory cytokines, such as TNF-α, IL-6, and nitric oxide (NO). To elucidate the underlying mechanism, we conducted further investigations. Western blot analysis showed that TaY reduced the phosphorylation of key proteins (IKK-α/β, IκB-α,NF-κB (P65)) in the TLR4-NF-κB signaling pathway and inhibited the inflammatory response. Furthermore, molecular docking and molecular dynamic simulations suggested that TaY binds to the hydrophobic pocket of MD2 through hydrogen bonding and hydrophobic interactions, potentially competing with LPS for MD2 binding. Collectively, TaY is a promising candidate for the development of novel therapeutic strategies against inflammatory disorders.

## 1. Introduction

Inflammation is an immune response of the organism in response to irritation or infection, which is an important mechanism to maintain health and prevent infection [1,2,3,4]. However, excessive inflammation can damage the organism and further produce various diseases: asthma, arthritis, and atherosclerosis [3,5,6]. Macrophages play an important role in the pathogenesis of these diseases [7,8]. Among the external substances that stimulate inflammation, the most important is LPS, a lipopolysaccharide from the cell wall of Gram-negative bacteria that activates the toll-like receptor (TLR) 4 signaling pathway, which, in turn, activates the body’s inflammatory response and a series of downstream events [9].

LPS induces TLR4 dimerization by binding to the hydrophobic structural domain of the myeloid differentiation protein-2 (MD2) [10], thereby transducing the signal into the cell. This process is carried out through the MYD88-dependent and TRIF-dependent pathways, respectively. MYD88-dependent signaling mainly activates the inhibitory kappa B kinase (IKK) and mitogen-activated protein kinases (MAPK) pathways [11]. IKKα, IKKβ, and IKKγ form a complex that catalyzes the phosphorylation of IκB protein, leading to IκB protein degradation and promoting nuclear transcription factor-κB (NF-κB) nuclear translocation, and the transfer of NF-κB to the nucleus to regulate a variety of downstream inflammatory factors (TNF-α, IL-1β, IL-6, NO) and related genes (cyclooxygenase-2, COX-2; inducible nitric oxide synthase, iNOS) expression [11,12].

Therefore, developing anti-inflammatory drugs that target key proteins is very effective in the inflammatory pathway. Currently, the commonly used anti-inflammatory drugs are mainly non-steroidal anti-inflammatory drugs (NSAIDs) [13], which work by inhibiting COX which is the key enzyme responsible for the synthesis of prostaglandins and messenger molecules during inflammation [14,15]. However, the major issue with NSAIDS is the toxic side effects associated with long-term use [16,17,18]. Therefore, developing new anti-inflammatory drugs with high efficacy and low toxicity has become a topic of great interest.

Peptide drugs combine the advantages of small chemical molecules (<500 Da) and protein drugs (>5000 Da) because of their molecular weight [19]. Their small molecular weight allows them to be synthesized using chemical methods, which offer precise structure, easy quality control, and low production cost. Additionally, their therapeutic effects are similar to those of protein drugs, which have the advantages of low toxicity and high specificity [20,21]. These make peptide drug highly advantageous in the development of anti-inflammatory treatments.

In the previous study, we screened a peptide TaY (KEKKEVVEYGPSSYGYG), which demonstrated excellent anti-inflammatory activity by luciferase-based reporter gene cell screening. We utilized the RAW-NF-κB luciferase reporter gene to screen for compounds that can stimulate cellular immune modulation. In this process, we found that, although TaY showed a weak stimulatory effect on the signaling pathway, it significantly reduced the increase in luciferase activity induced by LPS stimulation (unpublished data). In order to investigate the anti-inflammatory mechanism of TaY, this study was conducted using the LPS-induced macrophage RAW264.7 inflammation model to reveal the molecular anti-inflammatory mechanisms of TaY.

## 2. Results and Discussion

Inflammation is a bioprotective response to microbial invasion that can cause cellular damage, in which pro-inflammatory mediators are released to promote disease symptoms [1,2]. LPS-mediated inflammation is the most common [22]. Macrophages, as an important immune cell type that responds to inflammation, produce a series of pro-inflammatory mediators including TNF-α, IL-6, IL-1β, NO, and PGE2 [7,23]. Excessive production of these pro-inflammatory mediators can damage local cells and further aggravate symptoms [24,25]. Therefore, inhibiting the production of pro-inflammatory mediators is an effective strategy to suppress inflammation.

### 2.1. TaY Reduced the Expression of Pro-Inflammatory Cytokines in LPS-Induced RAW264.7 Inflammation

**Figure 1:**
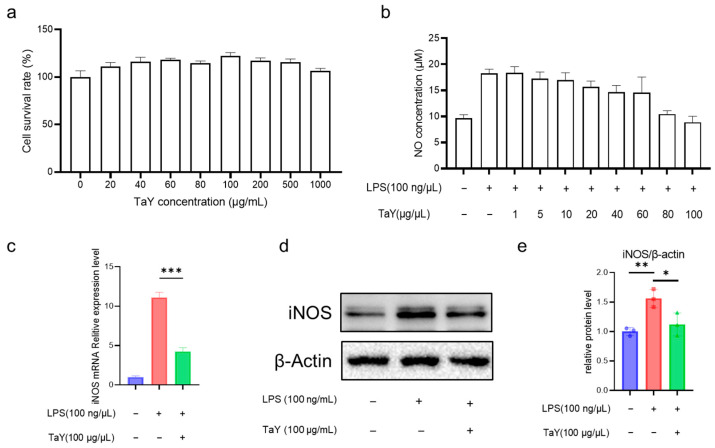
TaY reduces the NO level of LPS-induced RAW264.7 inflammation. (**a**) Cytotoxicity assay of TaY on RAW264.7; (**b**) effect of different concentrations of TaY on NO production; (**c**) TaY reduces the mRNA expression level of iNOS; (**d**) Western blot results of TaY reducing the expression of iNOS proteins, β-actin as the reference protein; (**e**) grayscale analysis of Western blot results. The data are presented as the mean ± SD (n = 3). NS, *p* > 0.05; * *p* ≤ 0.05; ** *p* ≤ 0.01; and *** *p* ≤ 0.001.

In this study, we first analyzed the toxicity of TaY on RAW264.7, and as shown in the Figure 1a, TaY was not significantly toxic to RAW264.7 in the concentration range of 20–1000 μg/mL. Nitric oxide, as an important marker of the inflammatory response, plays a very important role in several inflammatory diseases [26], and its ease of detection makes it a frequently preferred assay in the screening of anti-inflammatory drugs [27,28]. So we first analyzed the level of TaY to reduce LPS-induced NO production. As predicted, 100 ng/mL of LPS-stimulated cells resulted in a significant increase in NO levels, and the results showed that TaY’s ability to reduce NO levels exhibited a dose-dependent enhancement, which was almost the same level as that of the control group at 100 ng/μL (Figure 1b). These results demonstrated that TaY does, indeed, possess a strong anti-inflammatory ability. Additionally, we simultaneously examined the expression of iNOS, a major NO-producing gene. The results showed that TaY reduced the LPS-induced elevation of iNOS protein at both the transcriptional and protein levels (Figure 1c–e).

**Figure 2:**
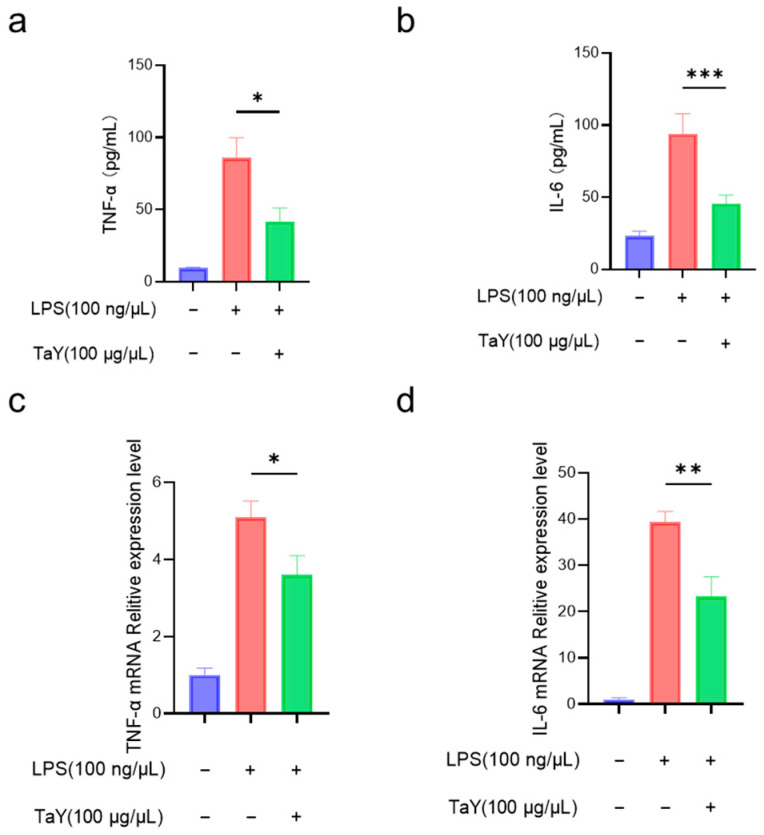
TaY reduces the proinflammatory cytokine of LPS-induced RAW 264.7 inflammation; (**a**,**b**) ELISA results of TNF-α and IL-6; (**c**,**d**) RT-PCR results of TNF-α and IL-6. The data are presented as the mean ± SD (n = 3). * *p* ≤ 0.05; ** *p* ≤ 0.01; and *** *p* ≤ 0.001.

The anti-inflammatory capacity of TaY was initially determined by the detection of NO, as well as the inducible enzyme, but in addition to NO, LPS stimulation caused an increase in the expression levels of several inflammatory factors (TNF-α, IL-6, and IL-1β) [11,12]. To further characterize the anti-inflammatory activity of TaY, we also evaluated the expression levels of the major pro-inflammatory cytokines TNF-α and IL-6 in RAW264.7 (Figure 2). As expected, the results were similar to those of NO and iNOS. TaY treatment was effective at decreasing the expression levels of pro-inflammatory factors, both at the transcriptional and protein levels (Figure 2).

Based on the level of reduction of LPS-induced pro-inflammatory mediators by TaY, its anti-inflammatory ability is quite strong, and peptide anti-inflammatory agents have been reported in many cases, but most of them do not have that strong anti-inflammatory activity compared to TaY. For example, Histatin-1 roughly reduces the level of LPS-produced NO by half [29], whereas TaY in the present study at 100 ng/μL could almost completely bring the level of NO production to the same level as the control (Figure 1b). This remarkable efficacy not only positions TaY favorably among peptide anti-inflammatory drugs, but it is also relatively rare among the remaining types of anti-inflammatory drugs reported [28,30,31].

### 2.2. TaY Inhibited the LPS-Activated TLR4-NF-κB Signaling Pathway

NF-κB, as a transcription factor, plays a crucial role in the expression of genes related to inflammation and immunity [32]. The TLR4-NF-κB signaling pathway is activated following LPS-induced inflammation in RAW264.7 [33]. TLR4 first activates MYD88, which recruits a series of downstream protein kinases to activate IKK. IKK then promotes the phosphorylation, ubiquitination, and subsequent protease-mediated degradation of IκB, allowing NF-κB to be released and translocated to the nucleus, where it functions as a transcription factor that promotes the transcription of a range of inflammation-related genes (iNOS, COX-2, TNF-α, IL-1β, and IL-6) [34,35].

**Figure 3:**
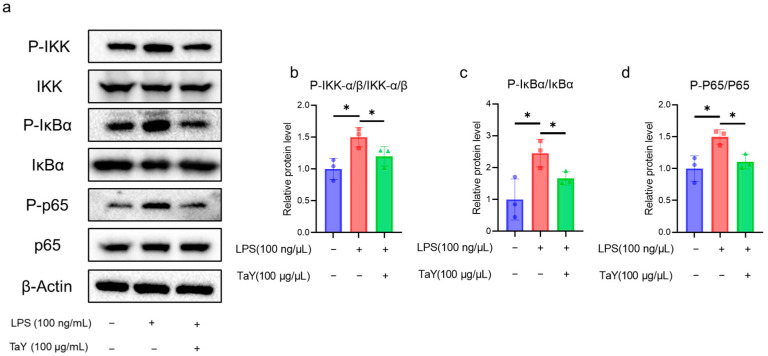
TaY inhibits LPS activation of the TLR4/NF-κB signaling pathway. (**a**) Western blot results for IKK, IκBα, P65 proteins, and their phosphorylated forms, with β-Actin as the reference protein; (**b**–**d**) grayscale quantitative results for the proteins of P-IKK-α/β/IKK-α/β, P-IκBα/IκBα, and P-P65/P65, respectively. The data are presented as the mean ± SD (n = 3). * *p* ≤ 0.05.

In the present study, we demonstrated that TaY reduced several inflammatory factors (TNF-α, IL-6, NO) in the LPS-induced inflammatory response. Next, we aimed to analyze the role of TaY in the TLR4-NF-κB signaling pathway. We first examined the protein expression levels in this pathway using Western blot. Previous studies have shown that LPS-induced NF-κB signaling is a dynamic process [36], so we first examined the dynamics of several key proteins in the NF-κB signaling pathway using our cells. The results indicate that iNOS expression increases over time, while p-IκBα expression peaks at 6 h and then begins to degrade. The expression of p-IKKα/β shows an increase from 3 h to 6 h, after which it is nearly indistinguishable from the control group. Similarly, p-p65 expression is also highest at 6 h. Based on the expression patterns of these proteins, we selected a 6 h LPS treatment duration for our formal experiments, and the results were shown in Figure 3. The results showed that TaY treatment resulted in a significant reduction in the phosphorylation levels of IKK-α/β (Figure 3b), followed by a decrease in the phosphorylation level of IκBα compared to the LPS-treated group (Figure 3c). This ultimately led to a reduction in the phosphorylation of P65 and decreased NF-κB entry into the nucleus (Figure 3d). These findings indicate a reduction in proinflammatory factors, including TNF-α, IL-6, and NO.

Through our Western blot experiments, we determined that TaY exerts its anti-inflammatory activity by inhibiting the TLR4-NF-κB signaling pathway. NF-κB is a critical node in the development of anti-inflammatory drugs, as activated NF-κB translocates to the nucleus to activate the expression of multiple inflammatory genes [34,35,37]. In this study, TaY achieves its anti-inflammatory function by regulating the TLR4-NF-κB signaling pathway. This mechanism is similar to that of another peptide, STM28, which inhibits downstream signaling by binding to TLR4 to exert anti-inflammatory activity [38]. Additionally, it has also been shown that peptide can exert its anti-inflammatory effects through the AMPK signaling pathway [29,39].

### 2.3. TaY Exerts Its Anti-Inflammatory Function by Binding to the MD2 Hydrophobic Pocket

In the previous study, we demonstrated that TaY exerts its anti-inflammatory function by inhibiting the activation of the TLR4-NF-κB signaling pathway, however, the specific mechanism by which TaY inhibits this signaling remains unknown. Peptide anti-inflammatory pathways have been reported to neutralize LPS and block signaling by inhibiting TLR4 and its ligands [40,41,42,43]. For example, LL-37, an antimicrobial peptide identified in the human body, exerts its anti-inflammatory effects by neutralizing LPS [44,45], while LK2(6)A(L), a peptide derived from the skin secretions of Chinese brown frog *Rana chensinensis*, can alleviate LPS-induced acute lung injury in mice by binding to MD2 [46]. The MD2 protein is essential for LPS function, as LPS must bind to the hydrophobic pocket of MD2 to activate TLR4 [47]. MD2-deficient mice are unresponsive to LPS stimulation, and some synthetic LPS-like chemicals, such as Eritoran, have been shown to inhibit LPS-induced inflammation [48,49].

**Figure 4:**
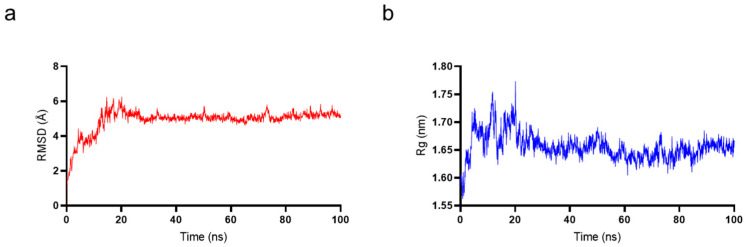
Molecular dynamics simulation results of TaY and MD2. (**a**) The root mean square deviation (RMSD) value for MD2 and TaY. (**b**) The radius of gyration (Rg) value for MD2 and TaY.

**Figure 5:**
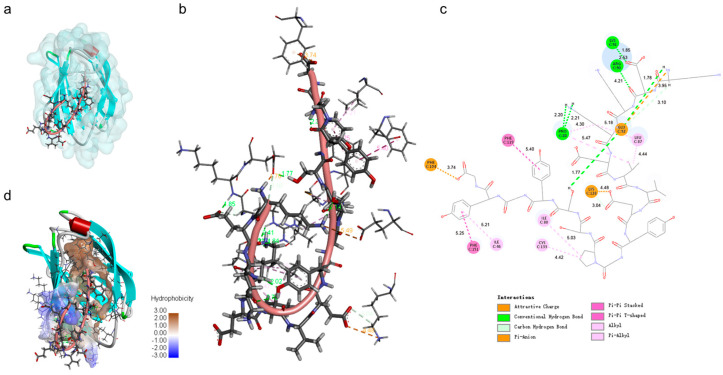
TaY can bind to the hydrophobic pocket binding domains of MD2. The overall 3D conformation (**a**), local interaction 3D conformation (**b**), and 2D conformation (**c**) of TaY docked to MD2 (**d**). The hydrophobic interaction surface demonstrating the results of the docking of TaY to MD2.

To determine whether TaY also exerts its anti-inflammatory function by competitively binding to MD2, we conducted molecular docking and molecular dynamics simulation to investigate the interaction between MD2 and TaY (Figure 4). The complex trajectory analysis showed a stable RMSD value at 5 Å after approximately 100 ns (Figure 4a), and the Rg value also stabilized at around 1.65 nm after the same duration (Figure 4b). We analyzed key interaction parameters between TaY and MD2, including electrostatic interactions, hydrogen bonds, and hydrophobic interactions (Figure 5b,c, Table 1). Current reports suggest that small molecules bind to residues Leu78, Asn86, Arg90, Glu92, Tyr102, Ser120, Phe121, Lys122, Ile124 of MD2 [47,49,50,51,52,53,54]. Our molecular docking results revealed hydrogen bonds between Lys1, Glu2, Lys4, Glu8, Ser13 of TaY and Pro88, Arg90, Lys91, Glu92, Lys128 of MD2. While Arg90, and Glu92 have been previously examined. Our findings indicate that Pro88, Lys91, and Lys128 also play significant roles in MD2 activation.

The binding of TaY to MD2 predominantly involves residues 1–9. Previous studies have shown that small molecules are crucial for hydrogen bond formation with residues located on the exterior of the hydrophobic pocket when binding to MD2 [49,55]. Our results similarly indicate that the portion of TaY forming hydrogen bonds with MD2 is primarily concentrated at the MD2 pocket site (Figure 5a,d). Additionally, residues 10–17 of TaY formed strong hydrophobic interactions with the hydrophobic core of the MD2 hydrophobic pocket (TaY: Pro11, Try14, Try16) (Figure 5c, Table 1). These hydrophobic interactions enhanced the binding of TaY to MD2, giving TaY a competitive advantage over LPS in binding to the MD2 hydrophobic pocket. However, these results are based on simulations and we cannot provide a definitive conclusion that TaY exerts its anti-inflammatory activity by binding to MD2. In the future, we still need to validate the interactions between TaY and MD2 based on the results of molecular docking in the experiment.

## 3. Materials and Methods

### 3.1. Synthesis of Peptides

Peptides were synthesized using the standard Fmoc solid-phase method by GL Biochem Ltd. (Shanghai, China). The purity of the peptides was 95%, and their relative masses were determined by MALDI-TOF-MS.

### 3.2. Cell Culture

Mouse macrophages (RAW264.7) were purchased from the Shanghai Cell Bank, Institute of Cell Biology, Chinese Academy of Sciences, and cultured in Duchenne’s Modified Eagle’s Medium (DMEM; HyClone, Utah, USA). The DMEM was supplemented with 10% (*v*/*v*) fetal bovine serum (Procell, Wuhan, China) and 1% (*v*/*v*) penicillin/streptomycin (Solarbio, Beijing, China), and incubated at 37 °C in a humidified environment (5% CO_2_, 95% air).

### 3.3. Cytotoxicity Analysis

The toxicity of TaY on RAW264.7 cells was assessed using the CCK-8 kit. RAW264.7 cells were seeded into 96-well plates at a density of 3.0 × 10^5^ cells per well and cultured for 12 h. After 12 h, the cells were treated with different concentrations of peptides (0, 20, 40, 60, 80, 100, 200, 500, and 1000 μg/mL) for 24 h. Subsequently, 10 μL of CCK-8 solution was added to each well according to the kit instructions and the cells were incubated for 2 h at 37 °C in the dark. The absorbance of the reaction solution was measured at 450 nm, and cell viability was calculated using the following formula:

$$Cell\;viability\;(\%)=\frac{A_{S}-A_{B}}{A_{C}-A_{B}}\times 100\%$$

*A_S_* is the absorbance of the well containing cells, CCK-8, and peptides; *A_B_* is the absorbance of the well containing medium and CCK-8, but without cells; and *A_C_* is the absorbance of the well containing cells and CCK-8, but without peptides.

### 3.4. Determination of Nitric Oxide Content

The LPS-induced inflammatory macrophage model was performed according to our previously described methodology [56]. LPS from *E. coli* 0111:B4 was purchased from Sigma-Aldrich (St. Louis, MO, USA). RAW264.7 cells were cultured in 96-well plates at a density of 3.0 × 10^5^ cells per well for 12 h. After 12 h of incubation, different concentrations of peptides (0, 1, 5, 10, 20, 40, 60, 80, and 100 μg/mL) were added to each well and incubated for another 3 h. Then, LPS was added to each well at a final concentration of 100 ng/mL and incubated for 24 h. The cell culture supernatants were used for the NO assay. NO levels were quantified using Griess reagents according to the manufacturer’s instructions (Beyotime, Beijing, China). Specifically, 50 μL of Griess A and 50 μL of Griess B were sequentially added to 50 μL of culture medium supernatant, and the absorbance of the reaction mixture was measured at 540 nm.

### 3.5. Enzyme-Linked Immunosorbent Assay (ELISA)

RAW 264.7 cells were cultured in 6-well plates at a density of 2.0 × 10^6^ cells per well for 12 h. After 12 h of incubation, 100 μg/mL of TaY was added to each well and incubated for another 3 h. Then, LPS was added to each well at a final concentration of 100 ng/mL and incubated for 24 h. After 24 h of incubation with LPS, cell supernatants were collected and then assayed using ELISA kits (Solarbio, Beijing, China) for IL-6, and TNF-α according to the manufacturer’s instructions.

### 3.6. RNA Isolation and Quantitative Real-Time PCR

RAW 264.7 cells were cultured in 6-well plates at a density of 2.0 × 10^6^ cells per well for 12 h. After 12 h of incubation, 100 μg/mL of TaY was added to each well and incubated for another 3 h. Then, LPS was added to each well at a final concentration of 100 ng/mL and incubated for 24 h. The cell pellets were the collected for RNA isolation. RNA isolation: total RNA was isolated using TRIzol reagent (Solarbio, Beijing, China), and the RNA integrity of all samples was assessed by agarose gel electrophoresis and NanoDrop. All cDNAs were synthesized using the HiScript^®^ III 1st Strand cDNA Synthesis Kit (Vazyme, Nanjing, China) according to the manufacturer’s instructions. Table 2 lists the primers used in this study. A two-step amplification method was used in this study: preincubation at 95 °C for 30 s, denaturation at 95 °C for 5 s, and extension at 60 °C for 30 s for a total of 40 cycles, followed by 95 °C for 5 s, 60 °C for 60 s, and melting at 95 °C for 1 s for a total of 1 cycle. The gene expression was normalized by comparing the expression of the target gene with that of the housekeeping gene β-actin. Results are reported as fold increase in gene expression relative to control samples.

### 3.7. Western Blot Analysis

RAW 264.7 cells were cultured in 6-well plates at a density of 2.0 × 10^6^ cells per well for 12 h. After 12 h of incubation, 100 μg/mL of TaY were added to each well and incubated for another 3 h. Then, LPS was added to each well at a final concentration of 100 ng/mL and incubated for 6 h. The cell pellets were then collected for protein extraction. Protein extraction was performed using RIPA, with protease inhibitors added proportionally, and protein concentrations were determined using the BCA method. Protein electrophoresis was performed using Tris-Gly SDS-PAGE, followed by membrane transfer using PVDF membranes. The membranes were blocket with 5% skim milk. The primary antibody for the target protein was incubated overnight. Then HRP-coupled secondary antibody was used to bind to the primary antibody, and the target protein was visulalized using a chemiluminescence visualizer. The following antibody were used in this study: p65 (1:5000), p-p65 (1:2000), IκB-α (1:5000), p-IκB-α (1:2000), IKK-α/β (1:5000), β-actin (1:10000) were purchased from Abmart (Shanghai, China); iNOS (1:2000) was purchased from proteintech (Wuhan, Hubei, China), p-IKK-α/β (1:2000) was purchased from Cell Signaling Technology (Beverly, MA, USA). The secondary antibody HRP-Goat Anti-Rabbit IgG (1:5000) was purchased from huaixngbio (Beijing, China).

### 3.8. Molecular Docking

The three-dimensional (3D) structure of the hybrid peptide TaY was built using PEPFOLD3.5 (https://bioserv.rpbs.univ-paris-diderot.fr/services/PEP-FOLD3/, accessed on 1 December 2016) [57]. The crystal structure of MD2 was obtained from Protein Data Bank (PDB ID: 2Z64). First, global rigid docking of TaY and MD2 was performed using HPEPDOCK (http://huanglab.phys.hust.edu.cn/hpepdock/, accessed on 2 July 2018) [58]. Local docking conformations were then optimized at the local server using ROSETTA’s FlexPepDock (http://flexpepdock.furmanlab.cs.huji.ac.il/, accessed on 27 May 2011), and the best docking conformations were selected based on scores [59]. Docking results were visualized using Discovery Studio 2021 and the interaction forces between the complex were analyzed.

### 3.9. Molecular Dynamics (MD) Simulations

GROMACS 2020.6 software was used to perform MD simulations of the YTP-TLR2 complex with the AMBER99SB-ILDN force field [60]. The complex was placed centrally in a dodecahedron box with a size of 1.2 nm and dissolved in water [46]. Na^+^/Cl^−^ ions were added to maintain the system in a neutral environment [46]. Energy minimization was performed using the steepest descent algorithm and continued until the maximum force was less than 1000 kJ/mol/nm, with a step size of 0.01 [61]. NVT and NPT ensembles were simulated using the leap-frog algorithm for 1 ns, with the temperature and pressure set to 310 K and 1 bar, respectively [62]. Finally, a 200 ns MD simulation was performed. After the stimulation, the trajectory file was analyzed using GROMACS, and the root mean square deviation (RMSD) and radius of gyration (Rg) of the system were obtained.

### 3.10. Statistical Analysis

Statistical analysis was performed using GraphPad Prism v9.0. Student’s *t*-test was used for statistical comparisons. All data are expressed as mean ± SD of at least three independent experiments. Significance was claimed with *p* values ≤ 0.05. NS: *p* > 0.05, *: *p* ≤ 0.05, **: *p* ≤ 0.01, ***: *p* ≤ 0.001, ****: *p* ≤ 0.0001.

## 4. Conclusions

In conclusion, our results suggest that TaY has promising potential as a peptide anti-inflammatory agent, which can reduce the activation of the TLR4-NF-κB inflammatory signaling pathway by LPS through the competitive binding of MD2 protein to LPS. The binding of TaY to MD2 reduces the phosphorylation levels of key proteins in the TLR4-NF-κB signaling pathway (IKK-α/β, IκBα), leading to decrease NF-κB nuclear translocation and ultimately a reduction in the expression of inflammatory factors (TNF-α, IL-6, NO, iNOS). These results demonstrate the potential of this peptide as a novel anti-inflammatory drug. In the future, we need to study the targets of TaY and MD2 to gain a deeper understanding of the anti-inflammatory mechanism of TaY.

## Figures and Tables

**Table 1:** Interactions between TaY and MD2.

Name	Distance	Category	From	To
B: Lys1:H1-C: Glu92:OE1	1.77	Electrostatic	B: Lys1:H1	C: Glu92:OE1
C: Lys128:NZ-B: Glu8:OE2	4.48	Electrostatic	C: Lys128:NZ	B: Glu8:OE2
B: Lys1:NZ-C: Glu122:OE1	5.49	Electrostatic	B: Lys1:NZ	C: Glu122:OE1
B: Gly17:OXT-C: Phe104	3.74	Electrostatic	B: Gly17:OXT	C: Phe104
C: Arg90:HE-B: Glu2:O	2.40	Hydrogen Bond	C: Arg90:HE	B: Glu2:O
C: Arg90:HH21-B: Glu2:O	1.83	Hydrogen Bond	C: Arg90:HH21	B: Glu2:O
C: Lys91:H-B: Glu2:OE1	1.84	Hydrogen Bond	C: Lys91:H	B: Glu2:OE1
B: Lys4:HZ2-C: Pro88:O	2.19	Hydrogen Bond	B: Lys4:HZ2	C: Pro88:O
B: Lys4:HZ3-C: Pro88:O	2.21	Hydrogen Bond	B: Lys4:HZ3	C: Pro88:O
B: Ser13:HG-C: Glu92:OE1	1.77	Hydrogen Bond	B: Ser13:HG	C: Glu92:OE1
C: Arg90:HA-B: Glu2:OE1	2.53	Hydrogen Bond	C: Arg90:HA	B: Glu2:OE1
C: Lys128:HE1-B: Glu8:OE1	3.03	Hydrogen Bond	C: Lys128:HE1	B: Glu8:OE1
B: Lys1:HA-C: Glu92:OE1	3.09	Hydrogen Bond	B: Lys1:HA	C: Glu92:OE1
C: Phe151-B: Tyr16	5.25	Hydrophobic	C: Phe151	B: Tyr16
C: Phe119-B: Tyr14	5.40	Hydrophobic	C: Phe119	B: Tyr14
C: Pro88-B: Lys4	4.30	Hydrophobic	C: Pro88	B: Lys4
C: Pro88-B: Val6	5.46	Hydrophobic	C: Pro88	B: Val6
C: Cys133-B: Pro11	4.41	Hydrophobic	C: Cys133	B: Pro11
B: Lys4-C: Leu87	5.17	Hydrophobic	B: Lys4	C: Leu87
B: Val6-C: Leu87	4.43	Hydrophobic	B: Val6	C: Leu87
B: Pro11-C: Ile80	5.03	Hydrophobic	B: Pro11	C: Ile80
B: Tyr16-C: Ile46	5.20	Hydrophobic	B: Tyr16	C: Ile46

**Table 2:** Sequences of the primers used for RT-PCR assays.

Gene		Sequence (5′-3′)	Length
*Tnf-* *α*	F	GGCCAACGGCATGGATCTCAAA	22
	R	TAGCAAATCGGCTGACGGTGTG	22
*IL* *-1* *β*	F	AATCTCGCAGCAGCACATCAACA	23
	R	ACACCAGCAGGTTATCATCATCATCC	26
*Inos*	F	TGGAGCGAGTTGTGGATTGTCCTA	24
	R	GCCTCTTGTCTTTGACCCAGTAGC	24
*Il-6*	F	TCTTGGGACTGATGCTGGTGA	21
	R	TTGGGAGTGGTATCCTCTGTGAA	23
*β* *-Actin*	F	TCACTATTGGCAACGAGCGGTTC	23
	R	CAGCACTGTGTTGGCATAGAGGTC	24

## Data Availability

Data are contained within the article.
